# Selected Properties of Densified Hornbeam and Paulownia Wood Plasticised in Ammonia Solution

**DOI:** 10.3390/ma15144984

**Published:** 2022-07-18

**Authors:** Przemysław Mania, Karol Hartlieb, Grzegorz Mruk, Edward Roszyk

**Affiliations:** Department of Wood Science and Thermal Techniques, Faculty of Forestry and Wood Technology, Poznań University of Life Sciences, Wojska Polskiego 38/42, 60-627 Poznań, Poland; karol.hartlieb@up.poznan.pl (K.H.); grzegorz.mruk@up.poznan.pl (G.M.); edward.roszyk@up.poznan.pl (E.R.)

**Keywords:** densified wood, ammonia, mechanical properties, Brinell hardness, Paulownia, hornbeam

## Abstract

The aim of the study was to densify samples of Paulownia Clone wood in vitro 112 and hornbeam (*Carpinus betulus* L.) by compression in the radial direction. Before the specimens were densified, they were subjected to plastic treatment in an ammonia solution. After densification, the compressive strength in the radial direction and the determination of the Brinell hardness in all three anatomical directions of the wood were determined. The wood swelling in humid air (98% RH) and liquid water was also determined. Paulownia wood density increased by about 280% and hornbeam wood density by 40%. The Brinell hardness parallel to the fibres increased by 49 and 390%, perpendicular by 80 and 388% for hornbeam and Paulownia, respectively. A significant increase in the compressive strength of wood in the radial direction was also observed. Densified hornbeam wood exposed to water showed a high swelling value of 153, while Paulownia wood exhibited 107%.

## 1. Introduction

It is known that the density of wood is related to its mechanical properties, i.e., the strength of the wood increases proportionally with the increase in its density. Densification, i.e., the thickening of the wood structure, improves the mechanical properties of low-density species and replaces hard species. Wood species with high hardness can also be densified, and their properties are even more improved [1]. The first concept of wood compaction appeared in 1900, but it consisted only of wood compression and did not consider plasticising treatment. Research on increasing wood density has been carried out for many years. It is said that this effect is achieved by compressing the wood in the transverse direction, which improves its mechanical properties [2,3,4,5,6]. In the 20th Century, patents for densification through wood compression began to appear [1,7]. These ideas involved mechanically compressing the wood tissue after subjecting it to steam under high pressure. However, this method did not capture the “memory effect” of wood, i.e., the post-deformation effect [8,9], which led to the “desire” for the wood to return to its original dimensions. According to Jakes et al. [8], hemicelluloses are responsible for dimension stabilisation of lignocellulosic materials. Lignin is the substance responsible for the shape memory effect. Delignification, leading to a reduction in the content of the components that make up the matrix encrusting the cellulose skeleton (hemicellulose and lignin), causes a significant decrease in post-deformation recovery because the hemicellulose in the wood is responsible for shape stabilisation. Lignin plays a substantial role in the quasi-elastic recovery mechanism [6,10,11,12,13]. Wood subjected to densification treatment has better physical and mechanical properties, but the increase in density does not always go hand in hand with improving these properties. This is permanent damage to the cell wall during such significant deformation of the wood [3,14].

The thermo-hygro-mechanical (THM) wood thickening process depends on the type of wood, both in terms of the degree of densification and the properties of the modified wood. This is due to the structural diversity and composition of the chemical cell walls of individual types of wood. Species with a lower density can be densified more easily than species with a higher value [15,16]. This is related to porosity and susceptibility to deformation. For this reason, when the timber is compressed in the radial direction, the earlywood zones first deform, and then the latewood zones. In this case, deformations of the early and latewood zones are inversely proportional to the value of their modulus of elasticity. Usually, the compaction of the wood tissue contributes to the improvement of the mechanical properties of the wood, even more than the increase in the density of the wood. Sometimes, however, the increase in the value of the mechanical parameters is smaller than the increase in wood density caused by its compaction.

One method that ensures plasticisation and fixation in one step is treatment with ammonia. Ammonia penetrates deeper into wood than water. This causes significant plasticisation of the wood, and the permanent displacement of particles becomes possible as the cellulose changes its morphology as new bonds are formed. The effect of ammonia is to swell the wood tissue, dissolve the low-molecular substances it contains, and split higher polymerised hemicelluloses, which is manifested by increasing the solubility of wood in water. Part of the ammonia is permanently bound to the lignin. Along with the destruction of hemicelluloses, some changes occur in the cellulose itself, and some of the bonds between lignin and carbohydrates are broken. Ammonia penetrates not only amorphous areas, but also, it can be assumed, partially in ordered areas. Due to the washing out of soluble substances and the deep penetration of ammonia and water particles into the cell membrane structure, an additional capillary system appears, and the inner surface of the wood is increased. Microfibrils gain greater freedom of movement from each other, manifested externally in the advanced plasticisation of the wood tissue [17,18,19,20,21].

*Paulownia Clone* in vitro, commonly known as Oxytree, is a new species that has appeared in Poland for several years. Apart from the tree’s physical and mechanical properties and the environmental impact of the tree, the species has not been thoroughly researched. Furthermore, not many studies related to the compression of this wood structure have been carried out. The aim of the study was to densify wood after its plasticizing in ammonia solution and to determine its selected properties. It was decided to determine the hardness of the wood in all anatomical directions and the compressive strength in the direction of densification. The dimensional stability of the material in water and humid air was also determined. Therefore, it was decided to compare the densification process after the wood was plasticised with ammonia on two completely different types of wood: the heaviest type of wood naturally occurring in Poland and one of the lighter ones, which is popular in Poland. For this purpose, hornbeam and Paulownia wood were used.

## 2. Materials and Methods

**Figure 1:**
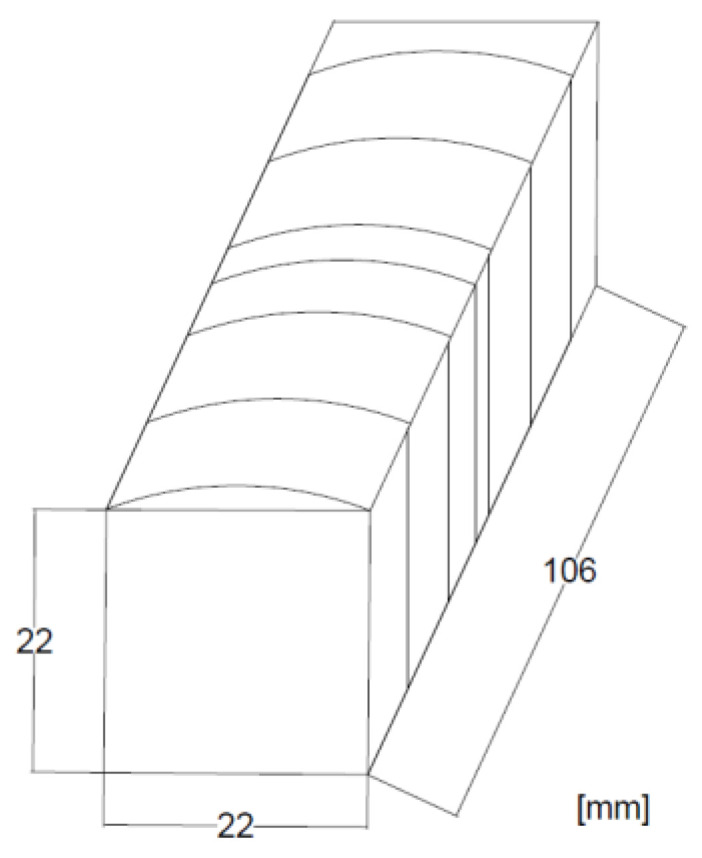
Sample shape and its dimensions.

The test samples were obtained from 10 Paulownia wood planks, 24 mm thick, purchased from a local seller, originating from Shantung Province (China). The boards were machined and cut into samples with lengths: of 22 mm in the tangential and longitudinal directions and 106 mm in the radial direction. The panels were selected so that the growth rings were as parallel as possible to the longer edge of the specimen. The moisture content (MC) of the samples obtained was about 12%. Hornbeam wood was the second experimental material, as hornbeam is the heaviest species that grows naturally in Poland. The material for the tests was taken from 60 mm-thick hornbeam logs. The log was cut into one-meter sections and mechanically processed to obtain samples of the exact dimensions as above. The drawing showing the model cut out with the dimensions marked is shown below (Figure 1). The densification process was applied to 70 samples of hornbeam and Paulownia wood. The exact number of samples was used as the reference material. Thirty samples were then used for the hardness determination, 30 for the compressive strength of the wood, and 10 for thickness swelling.

The density of the samples was determined before densification and after the modifications made to measure the degree of compaction as a percentage. Density was determined using the ISO 13061-2 standard [22]. Rectangular samples were measured in three directions with a calliper with an accuracy of 0.01 mm and then weighed on a laboratory balance with a measurement accuracy of 0.001 g. Density was calculated as the ratio of its mass and volume.

The samples were plasticised in a 25% ammonia solution. It consisted of heating the pieces in a boiling aqueous 25% ammonia solution for seven hours. The samples were placed in 6 L round-bottomed flasks. The flasks containing 2.5 L of the ammonia solution and the pieces were connected to a reflux condenser and placed in a heating mantle to bring the solution to a boil. The boiling point of the solution was 110 °C. After 7 h, the samples were taken out of the solution and inserted into the clamps in a hot-plasticized state.

**Figure 2:**
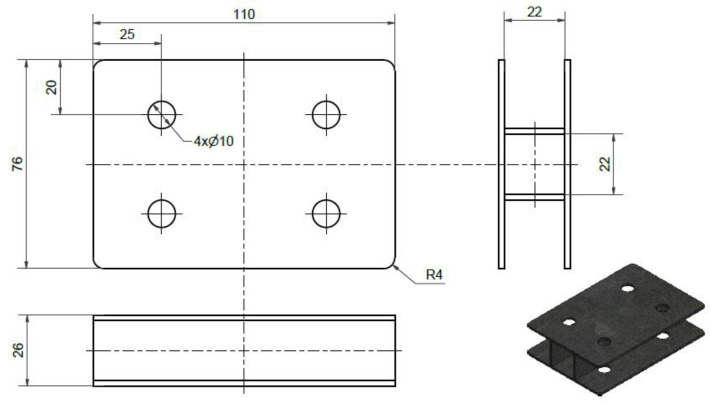
Drawing of the device for densification of wood samples.

A particular device was designed to densify the wood. The device was designed in such a way as to limit the possibility of the sample buckling on either side (Figure 2). Therefore, the samples after densification had a rectangular shape. Compression took place in the radial direction of the wood on a Zwick ZO50TH (Zwick/Roell, Ulm, Germany) strength testing machine. The maximum deformation that the samples could withstand without cracking and failure was 85 mm for paulownia and 55 mm for hornbeam wood samples. This compression of the samples was achieved by applying a stress of 2.5 N/mm^2^. This is the value beyond which the stress rapidly increased, as proven by preliminary tests. After pressing the specimens, the clamps were clamped by tightening the screws to prevent elastic recovery. The samples clamped in this way were left in the laboratory to dry.

After six weeks of conditioning, the densified wood samples were subjected to compression and a Brinell hardness test. The hardness of the tested materials was determined in three anatomical directions. A steel ball with a diameter of 10 mm was pressed into the surface of the samples with a force of 1000 N for densified and 500 N for control hornbeam samples and for paulownia wood, 500 N and 150 N, respectively. The maximum and minimum diameters of the residual indentation were measured with a Brinell magnifier to the accuracy of 0.1 mm. The hardness was calculated from the following formula:

(1) $$HB=\frac{2F}{D\pi \left(D-\sqrt{D^{2}-d^{2}}\right)}\;(\mathrm{MPa})$$

where *F* is the force acting on the ball (N), *D* is the diameter of the pressed ball (mm), and d is the average residual indentation (mm).

Compressive strength was determined in the direction of densification, so in the radial direction. The stress at the proportionality limit (so-called compressive strength perpendicular to the grain or relative strength (R_cR_ in the radial direction)) was determined. Compression tests were conducted using a numerically controlled test machine, Zwick Z050TH (Zwick/Roell, Ulm/Germany).

The maximum wood swelling degree was determined on five densified hornbeam and Paulownia wood samples. The maximum swelling was determined only in the radial direction. The oven-dried samples were measured in the radial direction with a calliper with an accuracy of 0.01 mm; then, the samples were placed in a desiccator above the water surface for 14 days (RH = 98%). After this time, the samples were again measured in this direction with an electronic calliper. Similar measurements were made on five consecutive samples, with the difference that the samples were placed in a beaker with distilled water for 14 days. Thanks to this, it is possible to evaluate the behaviour of such wood in humid air and water. The maximum swelling degree was calculated as the ratio of the increase in the size of the sample to its initial size.

The experimental data were analysed using the STATISTICA 13.3 software (TIBCO Software Inc., Palo Alto, CA, USA) with the analysis of variance (ANOVA). Significant differences between mean values of the parameters describing paulownia and hornbeam wood properties were determined using Tukey’s HSD test. The comparison tests were performed at a 0.05 significance level. Identical superscripts, e.g., a, b, c, denote no significant difference between mean values of the investigated properties.

## 3. Results and Discussion

The results of the determined density of hornbeam and Paulownia wood before and after densification are presented in Table 1.

As shown in Table 1, the average measured density of the hornbeam with a moisture content of MC = 12% is 786 kg/m^3^. This is a relatively low value for Poland’s heaviest and hardest native species because, according to Wagenführ [23], the average density of hornbeam is about 830 kg/m^3^ and the minimum is 530 kg/m^3^. The density at 790 kg/m^3^ was almost identical to [24,25]. The average density of the studied variety of Paulownia at 12% moisture is slightly lower than usual for this species. The density for Oxytree should be between 220 and 350 kg/m^3^ with an average value of 270 kg/m^3^ [26,27,28,29]. The average density for all densified samples of hornbeam wood increased by an average of about 320 kg/m^3^. However, this is only an average value. The densification results of individual specimens differed significantly, as evidenced by the difference between the minimum and maximum obtained density. The higher coefficient of variation for non-densified samples and the standard deviation show that the spread of density is significant compared to the control, unmodified wood. The density of hornbeam wood after densification increased by nearly 40% compared to the reference samples. A lower increase in hornbeam wood density can be explained by a higher initial density and a significant recovery of this wood after deformation. The densification process was carried out in the radial direction, where the wood rays constituted a substantial obstacle in the compaction process. The presence of aggregate wood rays characterises the hornbeam. However, the microstructure of these rays looks different than, for example, those in beechwood. The aggregate rays present in hornbeam wood are made of narrow wood rays separated by layers of wood fibres. A much higher increase in wood density was observed in Paulownia. The average value increased by over 2.8-times. A similar values, 740 kg/m^3^, was given by Li et al. [30] after densification. Although the increase in wood density is significant, it is still much lower than in the case of previously delignified wood [6,10]. It should be noted that the density of hornbeam and Paulownia samples was not the same. The initial height of hornbeam samples was reduced by 55 and Paulownia by 85 mm. The changes in wood density after the densification process were statistically significant for both types of wood.

Table 2 summarises the average values of the Brinell hardness for three anatomical directions (L—longitudinal, T—tangential, R—radial) of hornbeam wood and Paulownia wood before and after densification.

The table above shows that the wood has the highest hardness in the longitudinal direction, despite compacting it in the radial direction. The wood’s radial densification increases the wood’s hardness in three anatomical directions. Likewise, a significant change in hardness due to densification has also been reported for different densification processes [31,32,33]. The presented data indicate that the hardness of the tested materials is in the range of 5.63 MPa for control Paulownia wood to 103.35 MPa for densified hornbeam. The obtained results for the control hornbeam wood did not differ from the values given, for example, by [24,34]. Very similar results were also presented by Fodor et al. [35], where HB along the fibres was 67 and across the fibres 28 MPa. Comparing the hardness results for particular anatomical directions of hornbeam wood, a significant increase in this parameter can be noticed. The increase in hardness was about 80% perpendicular to the fibres and nearly 49% in the longitudinal direction in favour of densified wood. The lowest HB values characterised paulownia wood. In the longitudinal direction, it was only 10.6 MPa and across the fibres, only half of this value. There are no specific Brinell tests on Oxytree hardness in the literature. According to [26], the hardness determined by the Brinell method for the Paulownia tomentosa species is 19.7 MPa in the longitudinal direction, 9 MPa in the radial direction, and 8.2 MPa in the tangential direction, which is almost twice as high as in the Oxytree control samples. According to [36], HB parallel to fibres is about 27 and perpendicular 9.5 MPa. By densifying the Paulownia wood by 85 mm, the hardness increased nearly four-times in each anatomical direction. Li et al. obtained a minor increase in density with the densification of Paulownia wood, which amounted to 84 to 173%. The ANOVA analysis of variance confirmed that HB differed statistically after the densification process. Statistically insignificant differences occurred only in the hornbeam control wood, where the differences were statistically insignificant for both transverse directions.

Table 3 shows the average values and primary statistical data of the compressive strength of wood in the radial direction (R_cR_) carried out before and after the densification process.

The compressive strength of wood perpendicular to the grain is low. It is also impossible to determine the maximum compressive stress in the radial direction. During compression, successive wood zones are destroyed, resulting in the wood’s compaction. Therefore, stress at the limit of proportionality is most often taken as strength. Comparing the average values of the compressive strength of hornbeam wood samples before and after modification, a significant increase in this parameter can be noticed in the modified wood. On average, the compressive strength increased by almost 71%. The obtained results for the control hornbeam samples did not differ significantly from the results of other authors and fell within the given range of 9.7–11.5 MPa [25,37]. The Paulownia wood analysed had a very low compressive strength perpendicular to the fibres. During the densification of Paulownia wood and its shortening by 85 mm, the strength increased by over 320%. Such a large change may indicate significant changes in the structure of the wood. The force at the limit of proportionality was over 2100 N and was over three-times greater than the force in the control samples. The obtained differences are statistically significant at the significance level of 0.05.

**Figure 3:**
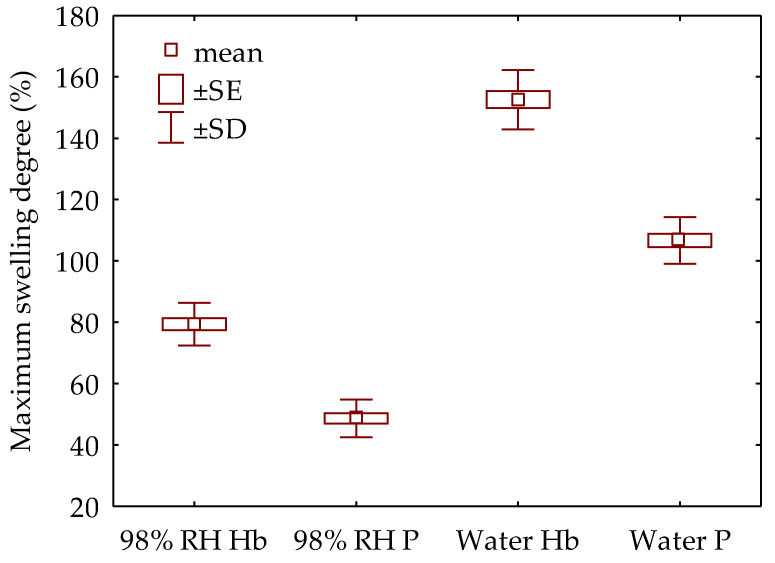
The maximum swelling degree of wood under various conditions (SE—standard error; SD—standard deviation).

A significant increase in the analysed mechanical parameters proves that ammonia is a suitable plasticiser. Its good plasticising properties have been described many times [17,19,21]. However, the biggest problem of mechanically densified wood is the post-deformation recovery associated with the discharge of the elastic energy previously accumulated and partially preserved in the drying process. Wood expansion deformations result from its elastic–viscous properties, exacerbated by moisture changes. In such a case, apart from the elastic deformations (immediate and delayed) resulting from the partial return of the deformed cell walls to their original form, there are also deformations related to the swelling of the wood. As the value of wood swelling increases with the greater density of wood, the problem of the moisture deformation of the densified wood is still the subject of many studies to reduce this unfavourable feature of such modified wood [8,9,38]. Therefore, it was purposeful to answer the question: How does the tested wood swell in the radial direction, i.e., in the direction of densification? Figure 3 shows the values of the maximum degree of swelling for densified hornbeam and Paulownia wood after 14 days of exposure to various humidity conditions. Both analysed materials behave similarly in conditions of increased humidity or liquid water. Hornbeam (Hb) wood was characterised by higher swelling. This is due to its higher density and aggregate wood rays. The wood swelling in humid air shows lower values. The process is more relaxed and slower. The post-deformation recovery of Paulownia (P) was about 50% and of hornbeam almost 80%. While in water, these values were about twice as high and about 107 and 153%, respectively.

Prior plasticisation of the wood in ammonia solution does not sufficiently stabilise the obtained material. Wood deformation recovery is significant. Jakes et al. [8] found that hemicelluloses are responsible for shape stabilisation and that lignin plays a significant role in the recovery mechanism. The chemical change in the wood after the action of ammonia is not substantial. Part of the ammonia is permanently bound to the lignin. Along with the destruction of hemicelluloses, some changes occur in the cellulose itself, and some of the bonds between lignin and carbohydrates are broken. Because lignin is not removed during the modification of ammonia, but is only combined with it, the recovery stabilisation effect is reduced. Partial removal of matrix components from the cell walls significantly reduces their relaxation effect. The above observation confirms the earlier results of [10].

## 4. Conclusions

The presented and discussed results led to the following conclusions:

1. Ammonia solution is a suitable wood plasticiser. The previously plasticised and mechanically densified hornbeam and Paulownia wood was characterised by a wood density higher than the control samples. This increase was approximately 40% and 280%, respectively.
2. Radial densification of wood contributed to a statistically significant increase in Brinell hardness in all anatomical directions of the wood. There was also a substantial increase in the compressive strength of wood in the radial direction.
3. The ammonia solution does not contribute to stabilising the shape of the obtained material in conditions of increased humidity. The recovery of samples immersed in water is essential.

## Figures and Tables

**Table 1:** Basic statistical parameters of the density of the studied wood species.

Material	Statistical Parameters				Coefficient of Variation, CV (%)
	$\rho_{min}$	$\rho_{mean}$	$\rho_{max}$	±SD	
	(kg × m^−3^)				
Control hornbeam	761	786 ^a^	831	31.5	4.01
Densified hornbeam	1010	1102 ^c^	1232	95.2	8.65
Control Paulownia	243	249 ^b^	279	6.3	2.53
Densified Paulownia	643	705 ^d^	784	59.2	8.39

^a–d^ different superscripts denote a statistically significant (*p* < 0.05) difference between mean values according to Tukey’s HSD test.

**Table 2:** Basic statistical parameters of the Brinell hardness (HB) of the studied material.

Material		Statistical Parameters		
		$HB_{L}\;(\mathrm{MPa})$	$HB_{T}\;(\mathrm{MPa})$	$HB_{R}\;(\mathrm{MPa})$
Control hornbeam	${\mathrm{HB}}_{\mathrm{mean}}$	69.24 ^d^	37.25 ^b^	38.13 ^b^
	±SD (MPa)	7.26	4.55	4.98
	CV (%)	10.48	12.21	12.82
Densified hornbeam	${\mathrm{HB}}_{\mathrm{mean}}$	103.35 ^c^	67.12 ^a^	65.64 ^e^
	±SD (MPa)	10.06	7.67	8.72
	CV (%)	9.73	11.42	13.28
Control Paulownia	${\mathrm{HB}}_{\mathrm{mean}}$	10.61 ^b^	5.63 ^d^	5.46 ^a^
	±SD (MPa)	1.22	1.06	1.10
	CV (%)	11.49	18.83	20.15
Densified Paulownia	${\mathrm{HB}}_{\mathrm{mean}}$	41.55 ^c^	22.51 ^e^	20.51 ^f^
	±SD (MPa)	5.49	2.39	2.63
	CV (%)	13.21	10.62	12.82

^a–f^ different superscripts denote a statistically significant (*p* < 0.05) difference between mean values according to Tukey’s HSD test.

**Table 3:** Basic statistical parameters of the compressive strength of the studied material.

Material	Statistical Parameters				Coefficient of Variation, CV (%)
	$R_{C_{R}\min}$	$R_{C_{R}\mathrm{mean}}$	$R_{C_{R}\max}$	±SD	
	(N × mm^−2^)				
Control hornbeam	10.11	11.75 ^c^	15.32	2.11	17.96
Densified hornbeam	18.23	20.08 ^a^	31.76	5.62	25.45
Control Paulownia	1.12	1.37 ^b^	1.65	0.20	14.82
Densified Paulownia	3.81	4.38 ^d^	5.45	0.75	17.05

^a–d^ different superscripts denote a statistically significant (*p* < 0.05) difference between mean values according to Tukey’s HSD test.

## Data Availability

All data are included in the text.
